# Meta-analyses of the neural mechanisms and predictors of response to psychotherapy in depression and anxiety

**DOI:** 10.1016/j.neubiorev.2018.09.022

**Published:** 2018-12

**Authors:** Lindsey Marwood, Toby Wise, Adam M. Perkins, Anthony J. Cleare

**Affiliations:** a Centre for Affective Disorders, Department of Psychological Medicine, Institute of Psychiatry, Psychology and Neuroscience, King’s College London, London, UK; b South London and Maudsley NHS Foundation Trust, London, UK; c Max Planck UCL Centre for Computational Psychiatry and Ageing Research, London, UK; d Wellcome Trust Centre for Neuroimaging, University College London, London, UK

**Keywords:** Depression, Anxiety, Neuroimaging, Psychotherapy, Meta-analysis, Functional

## Abstract

Understanding the neural mechanisms underlying psychological therapy could aid understanding of recovery processes and help target treatments. The dual-process model hypothesises that psychological therapy is associated with increased emotional-regulation in prefrontal brain regions and decreased implicit emotional-reactivity in limbic regions; however, research has yielded inconsistent findings. Meta-analyses of brain activity changes accompanying psychological therapy (22 studies, n = 352) and neural predictors of symptomatic improvement (11 studies, n = 293) in depression and anxiety were conducted using seed-based *d* mapping. Both resting-state and task-based studies were included, and analysed together and separately. The most robust findings were significant decreases in anterior cingulate/paracingulate gyrus, inferior frontal gyrus and insula activation after therapy. Cuneus activation was predictive of subsequent symptom change. The results are in agreement with neural models of improved emotional-reactivity following therapy as evidenced by decreased activity within the anterior cingulate and insula. We propose compensatory as well as corrective neural mechanisms of action underlie therapeutic efficacy, and suggest the dual-process model may be too simplistic to account fully for treatment mechanisms. More research on predictors of psychotherapeutic response is required to provide reliable predictors of response.

## Highlights

• Psychological therapies resulted in decreased activation in limbic regions: insula and ACC.
• Decreased prefrontal activation was also found pre-to-post psychological therapy.
• Results offer partial support for the dual-process model of psychotherapy.

## 1 Introduction

Psychological interventions are first-line treatments for depression and anxiety disorders (Baldwin et al., 2014; Cleare et al., 2015; NICE, 2009) but are ineffective for as many as 50% of patients (Cuijpers et al., 2014; Loerinc et al., 2015). Research investigating the neural correlates of therapy aims to provide a greater understanding about the formation, recovery and maintenance of symptoms, in addition to aiding the development of improved treatments and personalised medicine according to likely response (Lueken and Hahn, 2016), which could improve outcomes for recipients of psychological interventions. Recent reviews have highlighted the promise of functional neuroimaging studies in this field for both depression and anxiety disorders (Fu et al., 2013; Hamilton et al., 2012; Ma, 2015; Wise et al., 2014).

Neuroimaging studies take either a longitudinal approach, where patients are scanned before and after therapy, or a predictive approach where patients are scanned before therapy to determine pre-treatment brain-level predictors of subsequent symptomatic improvement. Longitudinal studies aim to identify changes in regional brain activity that are associated with the therapeutic mechanisms of the intervention. In contrast, prediction studies aim to provide a basis for stratified treatment according to likely response, potentially enabling clinicians to more effectively tailor therapies to individual patients (Fu et al., 2013). These complementary approaches may serve as a tool for clinical decision-making, along with behavioural markers gained from them.

Functional neuroimaging studies (using magnetic resonance imaging (MRI), single photon emission computed tomography (SPECT) or positron emission tomography (PET) scanning) typically demonstrate an imbalance in neural activation in patients with anxiety and depression compared to healthy controls whereby abnormally elevated limbic activation is not adequately controlled by prefrontal regions (Etkin, 2010; Hariri et al., 2000; Rauch et al., 2000; Whalen et al., 2002). These findings align with a dual-process model of emotion regulation with top-down prefrontal controlled processes and bottom-up, automatic limbic activation (Barrett et al., 2004). Prefrontal areas are involved in executive control (Owen et al., 2005) and emotional regulation processes (Ochsner and Gross, 2005), and have an inhibitory effect on limbic brain regions such as the amygdala, insula, hippocampus and anterior cingulate cortex (ACC), which are associated with intrinsic emotional reactivity (Drevets and Raichle, 1998; Phillips et al., 2003).

Patients with affective disorders who remit have been found to show recovery in the imbalance between these two systems (DeRubeis et al., 2008; Etkin et al., 2005; Siegle et al., 2007). DeRubeis et al. proposed that psychological therapies for depression act to regulate emotional control processes by increasing activation in prefrontal emotional regulation systems which in turn have a top-down effect on limbic activation (DeRubeis et al., 2008). An equivalent model in anxiety disorders has been proposed (Etkin et al., 2005).

There are, however, inconsistencies in the literature regarding the specific brain regions and direction of activation changes within regions (Goldapple et al., 2004; Linden, 2008) with some research being at odds with the model; for example, findings of decreased pre-frontal activation following psychological therapy (Taylor and Liberzon, 2007). Theoretically, this is not entirely unexpected as hyper-prefrontal activation has been associated with ruminative thinking (Goldapple et al., 2004) and the intrusiveness of traumatic memories (Lindauer et al., 2008) which would be expected to reduce with therapy.

For several reasons, findings from neuroimaging studies of treatment response may not be robust when considered independently. Results can be difficult to integrate comprehensibly especially given inconsistency in results and between study heterogeneity including differences in effect size caused by small sample sizes (Radua and Mataix-Cols, 2012). Meta-analyses in the field of neuroimaging provide an effective way to determine consistencies across datasets with improved statistical power.

### 1.1 Aims and hypotheses

The aim of this study was to use meta-analyses to determine the most robust changes with psychological therapy in two domains: 1) functional brain activation changes from before to after psychological therapy and 2) pre-treatment brain activation predictors of subsequent symptomatic improvement in patients with depression or anxiety disorders. To our knowledge this is the first prediction meta-analysis published in this field across both depression and anxiety related disorders. Both disorders were included due to high levels of comorbidity between the conditions (Brown et al., 2001; Kaufman and Charney, 2000), overlapping symptoms, both responding to similar therapies (Ressler and Mayberg, 2007), and the same theory being used to model therapeutic response across disorders (Messina et al., 2013). Further, meta-analyses across psychiatric disorders have found evidence of more similarities in functional and structural neuroimaging abnormalities across disorders than differences, despite variance in symptoms (Goodkind et al., 2015; McTeague et al., 2017). Additionally, all psychological therapies were considered due to evidence of commonalities between therapies such as therapeutic alliance, the opportunity to express thoughts and gain understanding of the self (Luborsky et al., 2002; Rosenzweig, 1936; Wampold et al., 2002).

Analyses were conducted using anisotropic effect-size seed-based *d* mapping (AES-SDM). This is similar to the more widely used neuroimaging meta-analytic technique called activation likelihood estimation (ALE, http://brainmap.org/ale); however, AES-SDM is able to provide a more accurate estimation of signal due to accounting for effect size in calculations (Radua et al., 2012). AES-SDM also has the advantage of permitting analysis of heterogeneity between studies via meta-regressions, and addresses between-study heterogeneity by counteracting the effects of studies reporting opposite activation findings in the same region by reconstructing both positive and negative maps in the same image (Radua et al., 2012).

We applied a thorough and conservative approach to identify the most robust findings within this heterogeneous literature. In line with the dual-process model, we hypothesised that psychological therapy would be associated with increased prefrontal activity and reduced limbic activity post- compared to pre-therapy. We hypothesised that increased baseline ACC activation would be predictive of greater symptomatic improvement in accordance with results from a meta-analysis primarily of pharmacological treatment prognostic neural biomarkers (Fu et al., 2013) and a recent review of neuroimaging predictors of response in anxiety and depression (Lueken and Hahn, 2016).

## 2 Methods and materials

### 2.1 Literature searches and study selection

Literature searches were conducted in the following electronic databases: Scopus (Elsevier, http://www.scopus.com), PubMed (https://www.ncbi.nlm.nih.gov/pubmed/) and Medline (Ovid Technologies Inc., http://ovidsp.uk.ovid.com) to identify articles published before 24.07.2017. The searches identified studies using MRI, SPECT or PET. All retrieved articles were evaluated for suitability. Reference lists of included articles and relevant reviews were manually searched.

The following eligibility criteria were applied:

• Articles were excluded if they did not include subjects meeting Diagnostic and Statistical Manual (DSM) or International Classification of Disease (ICD) (American Psychiatric Association, 2013; World Health Organization, 1993) diagnostic criteria for major depressive disorder (MDD); bipolar disorder; dysthymia; obsessive compulsive disorder (OCD); post-traumatic stress disorder (PTSD); panic disorder; social anxiety disorder (SAD); generalised anxiety disorder (GAD); or specific phobia (SP).
• Studies looking at the above affective disorders alongside neurological conditions were excluded to ensure findings were not obscured by neurological pathology.
• Participants were required to have been scanned prior to beginning a course of psychological therapy and have examined pre-treatment regional brain activation in relation to post-treatment change in symptom severity (prediction studies) or brain activity changes pre- to post-therapy (longitudinal studies). Studies were not excluded on the basis of concomitant psychotropic medication.
• Articles were excluded if they were case reports, reviews, meta-analyses, or not written in English.
• Only adult samples were suitable; studies focused on child, adolescent or geriatric populations were excluded to minimise the effect of neurodevelopmental and neurodegeneration confounders. In geriatric populations, there is an increased likelihood that organic disorders underlie, contribute to or confound depressive symptoms. Older patients are therefore likely to show age-specific neuroimaging correlates of therapy (Aizenstein et al., 2014; Smith et al., 2009). In adolescents, neurodevelopmental features need to be taken into account and inconsistencies have been found between adolescent and adult findings (Kerestes et al., 2014).
• Only whole brain results were included. Articles that used a region of interest (ROI) or machine learning approach only, did not apply consistent statistical thresholds throughout the brain (for example, regional resting-state analysis methods such as seed-based analyses), or did not report peak coordinates in stereotactic space were excluded.
• Both task-based and resting-state functional scanning paradigms were included. In order to control for any possible differences observed between these two study types, standard AES-SDM meta-analyses were conducted separately for task-based and resting-state studies and a meta-regression conducted controlling for paradigm type to increase methodological homogeneity where the number of studies permitted. This approach was taken as functional paradigm type can affect results and regions of activation (Fu et al., 2007; Messina et al., 2013; Palmer et al., 2015; Whitfield-Gabrieli et al., 2011).
• To ensure no overlap between studies, in the case of multiple studies reporting the same patient group, we included the largest sample or, in studies following up the same participant group at a range of time points post-therapy, the study reporting scanning at the time-point closest to therapy completion.

### 2.2 Meta-analyses

Analyses were carried out using AES-SDM (Version 5.141, 38). AES-SDM is a voxel-based, weighted meta-analytical method which creates voxel-level maps based on effect size and variance of peak-coordinates reported within studies and analyses them with random-effects meta-analytic methods. T-statistics are converted to effect sizes using standard statistical techniques. Effect size is calculated exactly at the reported peak coordinates and estimated, depending on distance from the peak, for the surrounding voxels using an anisotropic un-normalised Gaussian kernel multiplied by the effect size of the peak, subject to tissue-type constraints.

As suggested by Radua and Mataix-Cols (2012), voxels with a p-value <.005 were considered as significant, but those from clusters with fewer than 10 voxels or peaks with AES-SDM Z-values <1 were discarded to reduce the false positive rate. To determine the most robust results and explore the influence of outliers, a jackknife sensitivity analysis was conducted to assess the contribution of individual studies to the overall results. This repeats the analyses removing one study per iteration. Results were excluded that did not remain significant in 10% or more of iterations. To assess publication bias, funnel plots of effect size estimates of peak voxels were visually inspected and an Egger regression test implemented to examine funnel plot asymmetry (Egger et al., 1997). This was conducted using the metafor package for R software (Viechtbauer et al., 2010) (http://www.r-project.org/). The potential effect of paradigm type (task versus resting state scans) was examined by simple linear models and repeating standard AES-SDM meta-analyses in subgroups.

## 3 Results

### 3.1 Literature searches

**Fig. 1:**
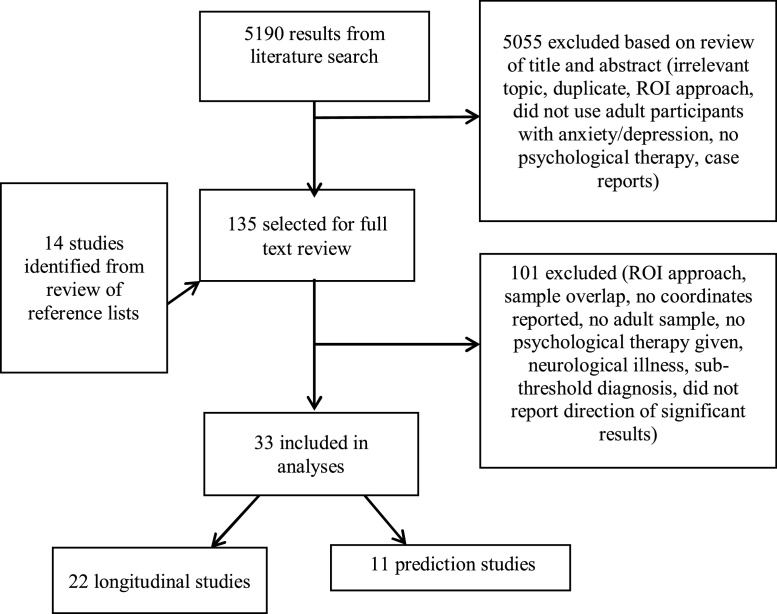
Flowchart of process of publication selection. Abbreviations – ROI, Region of Interest.

Scopus returned 3,559, PubMed 673 and Medline 958 results. From these, 33 articles were suitable for inclusion in analyses (see Fig. 1).

### 3.2 Longitudinal results

Twenty-two longitudinal studies (n = 352 patients) met eligibility criteria and were included in this analysis (see Table 1 for study details). The studies comprised the following patient groups: PD (n = 5); PTSD (n = 4); social anxiety disorder (n = 5); unipolar MDD (n = 3); SP (n = 2); OCD (n = 2); and GAD (n = 1). Disorder severity was typically in the moderate range (Table 1).

**Table 1:** Characteristics of longitudinal studies included in the meta-analyses.

Study	N (female)	Type of therapy	Imaging technique	Task	Severity	Medication	Comorbidities	Age (years)
Panic disorder (PD)								
Beutel et al., 2010	9 (6)	Psychodynamic therapy (4 week intensive inpatient program)	fMRI	Emotion regulation (emotional go/no-go task)	Met ICD-10 criteria for PD, 2 with agoraphobia, pre-treatment average score on agoraphobic cognitions questionnaire 2.04+-0.67	44.44%	Not stated	32
Kircher et al. (2013)	42 (29)	CBT (12 sessions)	fMRI	Fear conditioning	Met DSM-IV criteria for PD, PAS pre-treatment score 25.97, HAM-A 24.38	None	31 patients had 1 or more comorbidities. Excluded psychotic or bipolar I disorder, BPD.	35.42 (-)
Prasko et al. (2004)	6 (3)	Group CBT (6 weeks, 18 sessions, plus 2 individual booster sessions)	FDG PET	Resting state	Met DSM-IV criteria for PD with or without agoraphobia, mean pre-treatment PDSS score 16.5+-5.05	None	Patients with other axis 1 disorders were excluded	31.8 (-)
Sakai et al. (2006)	11 (9) all responders	CBT (10 sessions)	FDG PET	Resting state	Met DSM-III-R criteria, median pre-treatment PDSS score 16	None	Excluded: current MDD, bipolar, schizophrenia, social phobia, OCD, PTSD, GAD, personality disorder	29.26 +-6.39
Seo et al. (2014)	14 (10)	Group CBT (12 sessions)	Tc-99-ECD SPECT	Resting state	Met DSM-IV criteria for PD, average pre-treatment PAS score 24.86+-11.98	78.57%	Patients with other axis 1 disorders were excluded	32.3 +-9.02

Social anxiety disorder (SAD)								
Furmark et al. (2002)	6 (-)	Group CBT (8 sessions)	FDG PET	Symptom provocation (public speaking task)	Met DSM-IV criteria for social phobia (3 generalised)	None	None, excluded all other current psychiatric disorders	–
Goldin and Gross (2010)	14 (8)	MBSR (8 sessions)	fMRI	Emotional reaction to negative self-beliefs	Met DSM-IV criteria for SAD	None	Excluded all Axis 1 disorders except SAD, GAD, agoraphobia, or specific phobia	–
Goldin, et al. (2012)	24 (-)	MBSR (8 sessions)	fMRI	Self-referential encoding task (negative > self)	Met DSM-IV criteria for SAD (primary diagnosis)	None	Exclusion criteria included thought disorders, bipolar depression, and alcohol or drug dependence.	–
Klumpp, et al. (2013)	14 (9)	CBT (12 sessions)	fMRI	Emotional face processing (fearful versus happy)	Met DSM-IV criteria for SAD. Moderate to severe severity: pre-treatment LSAS 71.21+-9.61	14.29%	Excluded current MDD, severe depressive symptoms, history of bipolar or psychotic disorder	28.07 +-8.62
Månsson et al. (2013)	22 (-)	ABM (4 weeks, 8 sessions, n = 11), iCBT (9 week course, n = 11)	fMRI	Emotional face processing (disgust vs. neutral)	Met DSM-IV criteria for SAD, pre-treatment LSAS-SR: iCBT 76.00+-20.3; ABM 75.25+-19.2	36.36% ABM group, 45.45% iCBT group	Excluded current MDD	–

Post-traumatic stress disorder (PTSD)								
Aupperle et al. (2013)	14 (14)	Cognitive Trauma Therapy (mean 11.57 +-1.6 sessions)	fMRI	Emotional processing (anticipation and presentation of negative vs. positive images)	11 met full and 3 partial DSM-IV criteria, average pre-treatment CAPS score 66.07+-16.78	None	Excluded bipolar disorder of schizophrenia	40.07 +-7.44
Felmingham et al. (2007)	8 (5)	Imaginal exposure therapy and cognitive restructuring (8 sessions)	fMRI	Emotional face processing (fearful vs. neutral)	Met DSM-IV criteria for PTSD following assault (n = 4) or car accidents (n = 4)	25%	4 patients had comorbid MDD. Excluded psychosis and BPD	36.8 +-8.8
Lindauer et al. (2008)	10 (4)	Brief eclectic psychotherapy (16 sessions)	99mTc HMPAO SPECT	Symptom provocation (trauma scripts)	Met DSM-IV criteria for PTSD, pre-treatment PTSD score 11.7+-1.6	None	n = 3 mild depression. Excluded: schizophrenia, psychotic disorders, bipolar disorder, moderate and severe depression, panic disorder, phobia, OCD and dissociative disorders.	–
Pagani et al. (2007)	15 (-)	EMDR (5 sessions)	99mTc HMPAO SPECT	Symptom provocation (recollection of the traumatic event)	Met DSM-IV criteria for PTSD	None	Not stated	–

Major depressive disorder (MDD)								
Goldapple et al. (2004)	14 (-)	CBT (15-20 sessions)	FDG PET	Resting state	Met DSM-IV criteria for MDD, mean pre-treatment HDRS score 20+-3	None	Patients with other axis 1 disorders were excluded	–
Sankar et al. (2015)	16 (13)	CBT (16 sessions)	fMRI	Self-referential processing	Met DSM-IV criteria for MDD, average pre-treatment HDRS score 21.88+-1.89	None	Comorbid axis 1 disorders were excluded	40.00 +-9.27
Yoshimura et al. (2014)	23 (7)	Group CBT (12 sessions)	fMRI	Negative self-referential processing	Met DSM-IV criteria for MDD, average pre-treatment HDRS 11.0+-4.8	100%	Does not state (excluded psychotic disorder / bipolar)	37.3 +-7.2

Specific phobia (SP)								
Goossens et al. (2007)	16 (16)	Group CBT (exposure in vivo and modelling, 1 session, 4-5 hours)	fMRI	Symptom provocation (phobia vs. neutral images)	Diagnosed using the MINI (DSM), mean score on SPQ pre-treatment - 23.05+-2.88	None	Free from other lifetime history of psychopathology other than spider phobia	24 +-3.02
Schienle et al. (2007)	14 (14)	Group CBT/exposure (one 4 hour session)	fMRI	Symptom provocation (phobia vs. neutral images)	SPQ pre-treatment score: 21.9+-1.7	None	Not stated	27.2 +-9.2

Obsessive compulsive disorder (OCD)								
Baioui et al. (2013)	12 (8)	CBT (31 sessions)	fMRI	Symptom provocation (individualised OCD trigger photos)	Met DSM-IV criteria for OCD, pre-treatment YBOCS score 23.08+-12.63, illness duration at least 4 months	16.67%	4 patients had comorbid axis I disorders (2 SP; 2 MDD; 1 SAD)	32.49 +-8.89
Yamanishi et al. (2009)	33(19) responders only	Intensive behavioral therapy (12 weeks, sessions 1-5 times per week)	Tc-99-ECD SPECT	Resting state	Met DSM-IV criteria for OCD, average pre-treatment YBOCS score 33.5+-4.5	100%	Patients with other axis 1 disorders were excluded	34.7 +-7.1

Generalised Anxiety Disorder (GAD)								
Hölzel et al. (2013)	15 (9)	MBSR (8 weeks)	fMRI	Emotional face processing (angry versus neutral)	Met DSM-IV criteria for GAD.	20%	Comorbidities: N = 4 MDD, n = 5 SAD.	38.5 +-13.3

Missing data coded (-): PTSD, post-traumatic stress disorder: OCD, obsessive compulsive disorder: (g)SAD, (generalised) social anxiety disorder: PD, panic disorder: MDD, major depressive disorder: SP, specific phobia: FDG PET, fluorine-18-labelled deoxyglucose positron emission tomography: SPECT, single photon emission computed tomography: 99mTc-HMPAO, 99mtechnetium hexamethyl-propylene-amine-oxime :Tc-99-ECD, technetium-99m-ethyl cysteinate dimer: fMRI, functional magnetic resonance imaging: DSM, Diagnostic and Statistical Manual of Mental Disorders: ICD, International Statistical Classification of Diseases and Related Health Problems: LSAS, Liebowitz Social Anxiety Scale: PAS, Panic and Agoraphobia Scale: PDSS, Panic Disorder Severity Scale: HDRS, Hamilton Depression Rating Scale: HAM-A, Hamilton Anxiety Rating Scale; YBOCS, Yale-Brown Obsessive Compulsive Scale: STAI, State-Trait Anxiety Inventory: SPQ, Spider Phobia Questionnaire; EMDR, eye movement desensitisation and reprocessing therapy: ABM, attentional bias modification: (i)CBT, (internet-based) cognitive behavioral therapy: CAPS, Clinician-Administered PTSD Scale for DSM: MBSR, Mindfulness Based Stress Reduction.

**Fig. 2:**
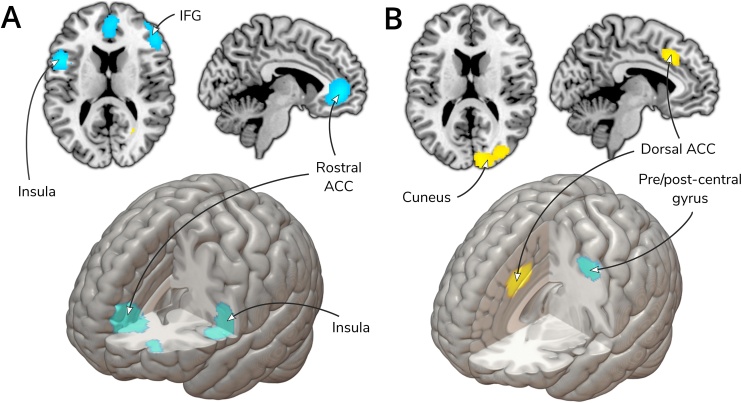
A) Results of longitudinal meta-analysis showing brain activation change pre-to post-treatment B) Results of prediction meta-analysis, regions predicting symptomatic improvement.

Table 2 provides details of all significant clusters from the longitudinal studies (n = 22). Details of jackknife sensitivity analysis, visual inspection of funnel plots, and publication bias analyses are detailed in the table. All regions survived sensitivity analysis and Eggers regression (all *ps*>.05), though some regions showed signs of publication bias in visual inspection of funnel plots. The most robust results, with no evidence of publication bias, were that psychological therapy was associated with significantly decreased activity post- compared to pre-therapy, in the left ACC/paracingulate gyri, the right inferior frontal gyrus and left inferior frontal gyrus/insula (all *ps*<.0001) (see Fig. 2).

**Table 2:** Regions of significant difference in brain activation change pre-to post-treatment.

Regions	Peak MNI coordinate	SDM Z-value	*p*	Number voxels	BA
**Neural activation: Post- > Pre-therapy**					
Right inferior network, inferior longitudinal fasciculus^1^	30, -62, -4	1.05	0.0007	118	–
Right arcuate network, posterior segment^2^	40, -54, 22	1.02	0.0008	82	–
Corpus callosum^1^	28, -62, 10	1.02	0.0008	19	–

**Neural activation: Post- < Pre-therapy**					
Left anterior cingulate / paracingulate gyri*	−2, 44, 4	−1.98	<0.0001	1548	10
Left inferior frontal gyrus, opercular part, left insula^3^	−50, 10, 14	−1.91	<0.0001	775	44
Right inferior frontal gyrus, triangular part*	48, 32, 20	−1.92	<0.0001	761	45
Left middle frontal gyrus^4^	−30, 52, 6	−1.30	0.001	101	10
Right temporal pole, middle temporal gyrus^5^	46, 4, -34	−1.20	0.002	64	20
Right middle frontal gyrus, orbital part^4^	26, 48, -14	−1.17	0.003	37	11

MNI, Montreal Neurological Institute; SDM, seed-based d mapping; BA, Brodmann Area. *Clusters surviving all tests of robustness and publication bias. 1 Driven only by two studies Goldin and Gross (2010) and Yamanishi et al. (2009) and funnel plots showed evidence of publication bias in this cluster. 2 Driven only by Yamanishi et al. (2009). 3 Driven by one study: Kircher et al. (2013). 4 Driven only by two studies: Goldapple et al. (2004) and Yamanishi et al. (2009) and a funnel plot showed evidence of publication bias in this cluster. 5 Driven by two studies: Kircher et al. (2013) and Prasko et al. (2004) and a funnel plot showed signs of publication bias in this cluster.

Too few studies met our eligibility criteria to perform meta-regressions to explore heterogeneity between disorders, concomitant medication status or therapy type (Radua et al., 2010). AES-SDM analyses were repeated and limited to task (n = 17) and resting-state studies (n = 5). The separate analyses showed that the clusters found overall (see Table 2) in the corpus callosum and left ACC/paracingulate gyri remained consistent across both subgroups (see Table 3, Table 4). The right inferior network (*p* = .0003, peak coordinates: 22, −60, −8), right arcuate network (*p* = .0004, peak coordinates: 40, −60, 20), bilateral inferior frontal gyri and right middle frontal gyrus (*p* = .00005, peak coordinates: 48, 34, 18) findings were only found in resting-state studies. Left inferior frontal gyrus (*p* < .0001 peak coordinates: -50, 10, 14) and left insula (which came as an additional separate cluster for task-based studies, *p* = .003, peak coordinates: −38, 0, −10) and right temporal pole/mid temporal gyrus (*p* = .003, peak coordinates: −46, 4, −34) were significant findings in task-based analysis only. This was confirmed with a linear model confirming significant differences in task versus resting-state in these regions.

**Table 3:** Regions of significant difference in brain activation change pre-to post-treatment– task-based studies only.

Regions	Peak MNI coordinate	SDM Z-value	*p*	Number voxels	BA
**Neural activation: Post- > Pre-therapy**					
Right and left precuneus / corpus callosum^1*^	6, -56, 38	1.19	0.0002	995	7/23

**Neural activation: Post- < Pre-therapy**					
Left inferior frontal gyrus, opercular part^2^	−50, 10, 14	−1.80	<0.0001	576	44
Left anterior cingulate / paracingulate gyri / right anterior cingulate ^3*^	−8, 44, -2	−1.67	0.0001	504	10
Left insula^2^	−38, 0, -10	−1.21	0.003	66	48
Right temporal pole, middle temporal gyrus^4^	−46, 4, -34	−1.21	0.003	64	20

Abbreviations - MNI, Montreal Neurological Institute; SDM, seed-based d mapping; BA, Brodmann Area. N=274. *Clusters surviving all tests of robustness. 1 Driven by one study: Goldin et al. (2012) and funnel plot showed signs of publication bias. 2 Driven by one study Kircher et al. (2013) and eggers regression test showed signs of publication bias. 3Driven by one study (Kircher et al., 2013). 4 Driven by two studies (Kircher et al., 2013; Heide Klumpp et al., 2013).

**Table 4:** Regions of significant difference in brain activation change pre-to post-treatment – resting-state studies only.

Regions	Peak MNI coordinate	SDM Z-value	*p*	Number voxels	BA
**Neural activation: Post- > Pre-therapy**					
Right lingual gyrus / right inferior network, right fusiform gyrus^1^	22, -60, -8	1.62	0.0003	686	19
Right arcuate network, posterior segment^1^	40, -60, 20	1.61	0.0004	232	–
Corpus callosum^1^	26, -64, 14	1.50	0.001	23	–

**Neural activation: Post- < Pre-therapy**					
Right middle frontal gyrus right inferior frontal gyrus *	48, 34, 18	−2.51	0.00005	949	45
Left middle frontal gyrus ^1^	−34, 56, 10	−2.30	0.00007	565	10
Right middle frontal gyrus, orbital part^1^	30, 46, -18	−2.31	0.00006	279	11
Right anterior cingulate / paracingulate gyri / left anterior cingulate^1^	4, 50, 12	−2.13	0.0002	250	32

MNI, Montreal Neurological Institute; SDM, seed-based d mapping; BA, Brodmann Area. N=78.

*Cluster surviving all tests of robustness. ^1^ Driven by one study (Yamanishi et al., 2009).

Regarding task-based longitudinal analysis, all but one region survived jackknife analysis criteria (the right temporal pole/middle temporal gyrus, *p* = .003, peak coordinates: −46, 4, −34, see Table 3). The most robust findings were the precuneus increased activation (*p* = .0002, peak coordinates: 6, −56, 38) and ACC deactivation (*p* = .0001, peak coordinates: −8, 44, −2) post-therapy, which showed no signs of publication bias.

None of the resting-state only clusters showed signs of publication bias (see Table 4). Due to few studies meeting eligibility for this analysis (n = 5), the only cluster meeting our criteria for robustness, surviving all iterations of the jackknife sensitivity analysis, was the right middle frontal gyrus (*p = .00005,* peak coordinates 48, 34, 18).

### 3.3 Prediction results

Eleven whole brain pre-treatment neuroimaging prediction studies (n = 293 patients) meeting eligibility criteria were included in this analysis (see Table 5 for study descriptions). All studies had analysed pre-treatment neural activation in relation to change in scores on measures of symptom severity. The studies comprised the following patient groups: PTSD (n = 2); SAD (n = 5); OCD (n = 2); MDD (n = 1) and PD (n = 1).

**Table 5:** Characteristics of prediction studies included in the meta-analyses.

**Study**	**N (female)**	**Type of therapy**	**Imaging technique**	**Task**	**Severity**	**Medication**	**Comorbidities**	**Age (years)**	**Prediction measure**
**Social Anxiety Disorder (SAD)**									
Burklund et al. (2017)	36 (-)	CBT (n = 17) or ACT (n = 19) both 12 sessions	fMRI	Dynamic social threat task (rejecting versus neutral phrases)	Met DSM-IV criteria for primary/co-primary SAD and clinical severity rating of >4/8	Some (-%)	Excluded bipolar disorder, substance-related disorders, suicidality, or psychosis.	–	Change in LSAS score
Doehrmann et al., (2013)	39 (14)	Group CBT (12 sessions)	fMRI	Emotional face processing (angry vs. neutral)	Met DSM-IV criteria for SAD, pre-treatment LSAS score of 81.8+-13.4	None	13 participants had comorbid anxiety disorders (6 GAD, 5 SP, 3 anxiety disorder NOS, PD 3, hypochondriasis 1)	29.3+-7.9	Change in LSAS score
Klumpp et al. (2014)	21 (15)	CBT (12 sessions)	fMRI	Emotional face processing (fearful/angry vs happy)	Met DSM-IV criteria for gSAD, moderate to severe severity: pre-treatment LSAS = 72.5+-11.6	9.52%	Excluded current MDD, severe depressive symptoms, history of bipolar or psychotic disorder, did not exclude comorbid anxiety disorders, SP (n = 3), GAD (n = 3), PD (n = 1)	24.9+-6.3	Change in LSAS score
Klumpp et al. (2016)	32 (24)	CBT (12 sessions)	fMRI	Emotional conflict resolutions (fear vs. neutral)	Met DSM-IV criteria for gSAD as primary complaint. Baseline LSAS 74.3+-14.9	None	Comorbid disorders not excluded: 10 GAD, 2 PD, 2 MDD, 4 dysthymia, 3 SP, 1 PTSD, 1 adjustment disorder	25.4+-5.1	Change in LSAS score
Klumpp et al. (2017)	34 (22)	CBT (12 session)	fMRI	Emotion regulation task (reappraise versus looking at negative images)	Met DSM criteria for SAD (primary diagnosis), moderate to severe: pre-treatment LSAS 77.7 +-14.0	None	Comorbid disorders not excluded: 11 GAD, PD 4, SP 3, PTSD 1, adjustment disorder 1	25.0 +-4.7	Change in LSAS score

**Post-traumatic stress disorder (PTSD)**									
Falconer et al. (2013)	13 (8)	CBT (8 sessions)	fMRI	Executive Inhibition (Go/No-Go task)	–	46.15%	Excluded history of psychosis or BPD. Comorbidities included: MDD (n = 8); PD (n = 1)	38.30+-12.16	Change in CAPS score
Aupperle et al. (2013)	14 (14)	Cognitive Trauma Therapy (mean 11.6 +-1.6 sessions)	fMRI	Emotional processing (anticipation and presentation of negative versus positive images)	11 met full and 3 partial DSM-IV criteria, score> = 30 on CAPS, average pre-treatment CAPS score 66.07+-16.78	None	Excluded bipolar disorder or schizophrenia	40.07+-7.44	Change in CAPS score

**Obsessive compulsive disorder (OCD)**									
Olatunji et al. (2014)	12 (6)	CBT (24 sessions)	fMRI	Symptom provocation	Inpatients. Met DSM-IV criteria for OCD, mean pre-treatment YBOCS 32.25(+-5.73)	66.70%	Excluded psychosis but no other axis 1 disorders were excluded. OCD had to be primary problem for which treatment was sought.	32.25 (range 18-53)	Change in YBOCS score
Yamanishi et al. (2009)	45 (26)	Intensive behavioral therapy (12 weeks, 1-5 sessions per week)	Tc-99-ECD SPECT	Resting state	Met DSM-IV criteria for OCD, average pre-treatment YBOCS score 33.81 (combined group mean calculated)	100%	Patients with other axis 1 disorders were excluded	34.01 (-)	Change in YBOCS score

**Major depressive disorder (MDD)**									
Carl et al. (2016)	33 (22)	BATD (mean 11.7+-4.4 sessions)	fMRI	Monetary incentive delay task (anticipation of reward)	Met DSM-IV-TR criteria for MDD, pre-treatments HDRS score of ≥15	None	Excluded current suicidal ideation, anxiety disorders, mood disorders other than unipolar depression or dysthymia, psychosis, substance disorders, past psychosis or bipolar disorder.	33.2 +-6.5	Change in BDI

**Panic disorder (PD)**									
Reinecke et al. (2014)	14 (10)	CBT (4 sessions)	fMRI	Emotion regulation	Met DSM-IV criteria for PD (8 with agoraphobia)	None	Comorbidities: 3 SP, 1 SAD. Excluded current or past psychotic disorder or bipolar.	37.2+-11.1	Change in ACQ score

Missing data coded (-): PTSD, post-traumatic stress disorder: OCD, obsessive compulsive disorder: (g)SAD, (generalised) social anxiety disorder: PD, panic disorder: Tc-99-ECD SPECT, technetium-99m-ethyl cysteinate dimer single photon emission computed tomography: fMRI, functional magnetic resonance imaging: DSM, Diagnostic and Statistical Manual of Mental Disorders: LSAS, Liebowitz Social Anxiety Scale: YBOCS, Yale-Brown Obsessive Compulsive Scale: CBT, cognitive behavioral therapy: CAPS, Clinician-Administered PTSD Scale for DSM: ACT, acceptance and commitment therapy: ACQ, Agoraphobia Conditions Questionnaire: BATD, Behavioral Activation Therapy for Depression: MDD, major depressive disorder: BDI, Beck Depression Inventory.

Only one cluster survived jackknife sensitivity analysis (a cluster with peak coordinates in the right cuneus cortex (*p* = .0004, peak coordinates: 10, −92, 14) which extended into the right superior occipital gyrus and right middle occipital gyrus); jackknife analysis revealed the other clusters were not robust (see Table 6). Evidence of publication bias was observed in all clusters’ funnel plots which was supported an Egger’s regression test with trend significance for the cluster of decreased activation (*t*(1, 10) = −2.17, *p* = .055) (See Fig. 2).

**Table 6:** Regions significantly predicting symptomatic improvement.

Regions	Peak MNI coordinate	SDM Z-value	*p*	Number voxels	BA
**Increased activity associated with greater symptomatic improvement**					
Right cuneus cortex^a^	10, -92, 14	1.74	0.0004	1066	18
Left median cingulate / paracingulate gyri / left anterior cingulate^2^	−4, 26, 36	1.81	0.0002	411	24
Right frontal orbito-polar tract^3^	20, 36, -14	1.66	0.0007	96	–

**Decreased activity associated with greater symptomatic improvement**					
Left precentral/postcentral gyrus^4^	−44, -2, 52	−1.09	0.0003	326	6

MNI, Montreal Neurological Institute; SDM, seed-based *d* mapping; BA, Brodmann Area.

a Driven by one study (Doehrmann et al., 2013). ^2^ Driven by two studies [65, 69]. ^3^ Driven by two studies (Klumpp et al., 2014; Yamanishi et al., 2009). ^4^ Not significant when two studies were excluded (Klumpp et al., 2014; Olatunji et al., 2014).

There were too few studies that met our eligibility criteria to perform meta-regressions (Radua et al., 2010) to study heterogeneity between disorders, therapies or methodologies (all but one study was task-based). When the meta-analyses was re-run on only studies which had used a task during scanning (n = 10), the four significant clusters as per the original analysis remained unchanged.

## 4 Discussion

These meta-analyses demonstrate that psychological therapy has robust effects on brain function and predicts therapeutic response across anxiety and depression, and provides partial support for the dual process model. Since the publication of similar reviews and meta-analyses (for example, (Brooks, 2015; Fu et al., 2013; Lueken and Hahn, 2016; Messina et al., 2013) the field has expanded rapidly, and the present study provides the largest and most up-to-date summary of the literature. Additionally, we used an improved analysis method which has various strengths compared to other neuroimaging meta-analytical techniques (Radua et al., 2014) and implemented a thorough and conservative approach to identify only the most robust studies within this heterogeneous literature fitting our eligibility criteria.

### 4.1 Longitudinal findings

The most robust findings were that psychological therapies resulted in decreased activation, post- compared to pre-therapy, in clusters with peak co-ordinates in the left ACC, inferior frontal gyrus (bilaterally) and left insula. It is important to note that studies had typically included both responders and non-responders in their analyses and therefore the changes are not indicative solely of treatment response. Due to our jackknife analyses, which indicated evidence of consistency in the findings across studies, the results appear to show brain activation changes which are consistent across psychological therapies and are trans-diagnostic. However, it is important to highlight that these findings do not signify that there are not activation changes that are specific to types of psychological therapy or able to differentiate between disorders and their subtypes. There were currently, however, too few studies to study disorder- or treatment-specific brain activation changes. Additionally, it would be difficult to confidently study one disorder in isolation from another due to high levels of comorbid Axis I disorders in the patient samples (see Table 1, Table 5).

The analyses were run separately on task and resting-state studies due to evidence that paradigm type can effect results (Fu et al., 2007; Messina et al., 2013; Palmer et al., 2015; Whitfield-Gabrieli et al., 2011). Our subgroup analysis revealed substantial differences between these paradigms; however, a decrease in ACC activity post-therapy was a common finding across both resting-state and activation paradigms. This result is in agreement with a recently published systematic review on brain activation changes with CBT summarising that the most consistent finding is decreased dorsal ACC activity (Franklin et al., 2016). This region is involved in both emotional processing and regulation and has been linked to self-referential processing and cognitive and attentional control with strong connections to both limbic and prefrontal brain regions (Pizzagalli, 2011).

We did not find dlPFC involvement despite this region also being associated with attentional control and emotional regulation (Hofmann et al., 2012; Kane and Engle, 2002; Owen et al., 2005; Wager and Smith, 2003). This could be due to an insufficient number of studies in our meta-analyses to demonstrate this effect and an inconsistency between the designs of included studies. We did however find significant effects elsewhere in prefrontal brain regions, including the IFG which is a key region involved in emotional regulation and inhibition (Aron et al., 2003, 2004) which suggests involvement of the PFC in affective disorders may be complex and not attributable to a single region (Fitzgerald et al., 2006; Thomas and Elliott, 2009).

### 4.2 Implications for the dual-process model

The dual-process model is appealing due to its parsimony and fitting with the theoretical modes of action we would expect from treatments for affective disorders. For example, CBT is proposed to improve emotional regulation by challenging negative cognitions and improving conscious emotional regulation. We would therefore expect greater cognitive control to be evident in prefrontal conscious emotional-regulation brain regions. The decreased activation we found in limbic regions (the ACC and insula) is consistent with this emotional regulation model of depression and anxiety. However, the decreased activation we found bilaterally in the inferior frontal gyrus runs counter to this, as the theory proposes increased activation in pre-frontal regions, associated with emotional regulation.

Despite these findings being at odds with the model, they do not necessarily undermine its credibility. Decreased prefrontal activity, particularly in resting-state studies, may signify an enhanced capacity for top-down regulation when required i.e., these areas were dysregulated but regained the capacity to respond appropriately and are ‘better’ utilised when necessary after psychological therapy.

However, the model may be too simplistic as it ignores any compensatory changes in functioning that may be occurring. This more complex model has been proposed by Willner et al. (2013) in relation to the mode of action of antidepressants, but we suggest that there are likely to be compensatory as well as normalising mechanisms involved with psychological therapies also.

Additionally, it is unlikely that the effects of psychological therapies can be solely represented by cognitive control and voluntary emotional regulation with a linear relationship between prefrontal and limbic regions. Messina et al. proposed an alternative neural model of action of psychological therapy, albeit with a focus on psychodynamic therapy models (Messina et al., 2016). They highlighted that the dual-process model ignores that psychodynamic therapy aims to regulate emotional states, not only by strengthening executive control but through the resolution of early childhood parental interactions and challenging negative representation of the self and others in relationships. They therefore postulated that one should expect direct changes in default mode network and implicit emotional regulation regions which are involved in self-referential processing. Their model may also be applicable to other psychological therapies which also place importance on challenging negative self-views.

### 4.3 Comparison to antidepressants

The neural effects of psychological therapy are vastly understudied compared to those of antidepressants. Ma conducted a meta-analysis of the neural correlates of antidepressants which included 60 studies (n = 1,569) (Ma, 2015) and found decreased activation in the ACC, amygdala and thalamus with antidepressant medication and increased activation in the dlPFC. These results fit the dual-process model which hypothesises that antidepressants act more directly on the emotional, limbic network whereas psychological therapies primarily target prefrontal function by increasing inhibitory executive function. However, we found evidence of reduced activity in limbic areas with psychological therapies and therefore differentiation between treatment modalities may be more complex than proposed. Further work directly comparing treatment modalities is required to explore how far changes reflect general as opposed to treatment-specific modes of recovery. Studies with a more frequent follow-up throughout the course of treatment would enable us to more rigorously test the dual-process model to determine whether differential primary actions between treatment modalities exist. Additionally, work using dynamic causal modelling of fMRI data or transcranial magnetic stimulation could further allow us to determine the causal direction of results.

### 4.4 Prediction findings

In terms of the prediction data, we found one area, the right cuneus cortex, whose greater activation at baseline was associated with greater symptomatic improvement. This extrastriate region has been implicated in response inhibition in particular those involving motor reactions (Booth et al., 2005; Matthews et al., 2005). The cuneus forms part of the DMN, which has been found to be abnormally activated in depression (Greicius et al., 2009). However, there is inconsistency between study results and too few published studies at present to determine further robust predictors of symptomatic improvement with psychological therapy. Speculatively, this could imply that prediction is more disorder or treatment specific but further work is required to test this.

We hypothesised that increased baseline ACC activation would be associated with symptomatic improvement in line with previous reviews (Fu et al., 2013; Lueken and Hahn, 2016). We did find that elevated left ACC activation was associated with greater symptomatic improvement; however, this region did not meet our criteria for robustness. Lueken and Hahn (2016) note in their systematic review that the direction of predictive effects of ACC activity was dependent both on the type of functional imaging paradigm used and on the specific psychological treatment received (Lueken and Hahn, 2016). Therefore, ACC activation could have been masked in this meta-analysis. Currently, however, there were too few studies to explore the effects of between-study heterogeneity on this analysis.

### 4.5 General strengths and limitations

Although these meta-analyses present a comprehensive summary of the evidence-base so far, the results should be considered cautiously. The present literature is small meaning the influence of between study heterogeneity, other than paradigm type, could not be assessed through meta-regressions.

Between study heterogeneity could have influenced the results of these analyses in several ways. Firstly, all functional neuroimaging designs were included ranging from resting-state to emotionally distressing or cognitively demanding tasks. Although we did control for resting-state versus task-based methodology to increase specificity in findings, even the type of task can have a great effect on the neural activation detected (Fu et al., 2007; Palmer et al., 2015). By adopting inclusive eligibility criteria for paradigm type, this will have increased power given the paucity of research in this field and allowed greater generalisability of global results to broad neurobiological models. Secondly, the included studies comprised patients with a range of disorders, comorbidities, and symptom severity, another source of between- and within-study variability. Anxiety disorders were over-represented compared to depression. Thirdly, we would expect that the specific neural changes occurring with therapy would differ according to the type of psychological therapy the patient received (for example as has been found with studies directly comparing different therapies (Burklund et al., 2017; Månsson et al., 2013). Additionally, the studies varied on the concomitant psychotropic medication status of the patients (see Table 1, Table 5) which reduced our ability to conclude that the neuroimaging effects are solely due to psychological therapy. However, most studies required patients to have been on the medication for an adequate trial (typically 6 or more weeks) and the medication to be kept stable for the duration of the study. Finally, we included SPECT, PET and fMRI scanning methodologies. These methods differ in their measurement of brain activity, including temporal and spatial resolution. Therefore, it is plausible that findings from the various modalities could differ considerably. However, all included PET and SPECT studies used radiotracers to measure regional brain glucose metabolism, which is the measurement most related to fMRI BOLD signal. Additionally, we only included studies where participants fulfilled diagnostic criteria. Although warranted given the scope of these meta-analyses the results may not be generalisable to all individuals who evidence subthreshold clinical anxiety or depression. Despite considerable heterogeneity, patients in the included studies were typically in the moderate to severe range of severity, most therapies were cognitive and/or behavioral in nature, and a negative emotional scanning paradigm was primarily used.

Another limitation is that we only included results of the patient group who received therapy. Care should be taken when considering the results of these meta-analyses, and indeed studies in this area, as effects are unlikely to be solely attributable to the treatment under investigation and may in part be due to spontaneous remission or concomitant therapy. This problem could be ameliorated by the inclusion of a placebo arm (for example, one-to-one non-therapy sessions or wait-list control groups). Although fully balanced designs, with control groups who also receive scans at both time points, are best practice in order to properly model the effect of repeated scans and other non-treatment related factors (Dichter et al., 2012), including only these studies was not within the scope of these meta-analyses in order to maximise the number of suitable studies.

Additionally, as with all meta-analyses, the potential influence of publication bias should be considered when interpreting results. Although, in our longitudinal meta-analysis, we did not show any evidence of this, there were signs of publication bias in the prediction of treatment response meta-analysis. Also, our reliance on including only peak co-ordinates reported in published papers does not provide the level of detail that statistical parametric maps or individual-level data would.

Despite considerable variability in study designs which these meta-analyses illustrate, commonalities did emerge, and we were able to demonstrate some consistent findings. In order to enhance the discovery of brain-biomarkers of response and therapeutic action, future studies should include larger samples and work to consistent study designs and analytical techniques.

In conclusion, our meta-analyses demonstrate that there are consistent brain activation changes in psychological therapy across depression and anxiety disorders, although the literature is relatively small and there is considerable between-study heterogeneity. However, neural changes that are robustly predictive of treatment response remain elusive. We suggest that more research is required to form definitive conclusions in order to benefit patients at an individual level by tailoring treatment according to likely response and understanding treatment mechanisms in order to improve treatments.

## Financial disclosures

AJC has in the last three years received honoraria for speaking from Astra Zeneca (AZ) and Lundbeck, honoraria for consulting from Allergan and Livanova and research grant support from Lundbeck. AMP is supported by Bionomics Limited. LM and TW report no conflicts of interest.
